# Role of nanoparticle size and sialic acids in the distinct time-evolution profiles of nanoparticle uptake in hematopoietic progenitor cells and monocytes

**DOI:** 10.1186/s12951-019-0495-x

**Published:** 2019-05-13

**Authors:** Bart Wathiong, Sarah Deville, An Jacobs, Nick Smisdom, Pascal Gervois, Ivo Lambrichts, Marcel Ameloot, Jef Hooyberghs, Inge Nelissen

**Affiliations:** 1 Health Department, Flemish Institute For Technological Research (VITO), Boeretang 200, 2400 Mol, Belgium; 2 Biomedical Research Institute (BIOMED), Hasselt University, Agoralaan Building C, 3590 Diepenbeek, Belgium; 3 Theoretical Physics, Hasselt University, Agoralaan Building D, 3590 Diepenbeek, Belgium

**Keywords:** Nanoparticles, Hematopoietic progenitor cells, Monocytes, Flow cytometry, Confocal imaging, Proteoglycans

## Abstract

**Background:**

Human hematopoietic progenitor cells (HPCs) are important for cell therapy in cancer and tissue regeneration. In vitro studies have shown a transient association of 40 nm polystyrene nanoparticles (PS NPs) with these cells, which is of interest for intelligent design and application of NPs in HPC-based regenerative protocols. In this study, we aimed to investigate the involvement of nanoparticles’ size and membrane-attached glycan molecules in the interaction of HPCs with PS NPs, and compared it with monocytes. Human cord blood-derived HPCs and THP-1 cells were exposed to fluorescently labelled, carboxylated PS NPs of 40, 100 and 200 nm. Time-dependent nanoparticle membrane association and/or uptake was observed by measuring fluorescence intensity of exposed cells at short time intervals using flow cytometry. By pretreating the cells with neuraminidase, we studied the possible effect of membrane-associated sialic acids in the interaction with NPs. Confocal microscopy was used to visualize the cell-specific character of the NP association.

**Results:**

Confocal images revealed that the majority of PS NPs was initially observed to be retained at the outer membrane of HPCs, while the same NPs showed immediate internalization by THP-1 monocytic cells. After prolonged exposure up to 4 h, PS NPs were also observed to enter the HPCs’ intracellular compartment. Cell-specific time courses of NP association with HPCs and THP-1 cells remained persistent after cells were enzymatically treated with neuraminidase, but significantly increased levels of NP association could be observed, suggesting a role for membrane-associated sialic acids in this process.

**Conclusions:**

We conclude that the terminal membrane-associated sialic acids contribute to the NP retention at the outer cell membrane of HPCs. This retention behavior is a unique characteristic of the HPCs and is independent of NP size.

**Electronic supplementary material:**

The online version of this article (10.1186/s12951-019-0495-x) contains supplementary material, which is available to authorized users.

## Background

Nanotechnology comprises the development, synthesis and application of materials of which size and shape are defined at the nanoscale [1]. Their dimensions and intrinsic physicochemical properties differentiate nanoparticles (NPs) from their bulk form. These specific characteristics drives the use of NPs in a wide range of applications such as electronics, pollution remediation, cosmetics and coatings [1]. NPs are reported to enter living cells and to interact with cellular processes with potentials in diagnostics [2] and therapeutics, for example as drug carriers [3]. Moreover, their high surface-to-volume ratio with corresponding increased surface reactivity, enables the NPs to interact efficiently with biological and chemical processes [4, 5]. Indeed, while the field of conventional medicine is facing various problems, e.g. drug toxicity, diminished target specificity and insufficient bioavailability, nanotechnology is expected to provide solutions for these issues [6]. However, nanoparticle-related safety concerns [7] due to non-specific cellular uptake and bioaccumulation issues [8] delay the commercialization of nanotechnology-based therapeutic applications. In cell-based tissue regeneration, NPs are being examined to guide or track human hematopoietic progenitor cells (HPCs) to their target location [9, 10], and monitor their engraftment and local differentiation process [11–13]. These nanotechnology-based approaches for detection and monitoring of cell transplants have been demonstrated to improve clinical cell therapy [14]. However, to make these applications available for use in patients, challenges with respect to safety and efficiency have to be conquered. In this respect, knowledge on the cellular processes at the outer membrane underlying the interaction between NPs and transplanted cells is essential.

The interactions between NPs and the cells at their outer membrane, is dependent on both the biological parameters, e.g. cell surface proteoglycans [15], and on the physicochemical properties of NPs, such as size [16, 17]. Alike all vertebrate’s cells, HPCs express glycan molecules at their outer membrane [18, 19]. These are negatively charged surface molecules and constitute a group of transmembrane glycoproteins that can have major influences in the interaction with extracellular structures and molecules [20]. These glycosaminoglycans (GAGs), such as sialic acids can also be found in the niche of HPCs [21, 22] were they play a crucial part in cell signaling. Through these signaling pathways, GAGs can modulate cellular processes such as self-renewal and differentiation [19]. GAGs are also of major importance in the interaction of cells with extracellular objects such as NPs. Multiple studies have already demonstrated the determining role of the dense endothelial glycocalyx on the uptake of NPs [23–25]. To our knowledge, there are no research data available demonstrating the involvement of membrane-attached glycan molecules in the interaction of HPCs with NPs. In our previous study [26], we observed a transient loading behavior upon exposure of HPCs to carboxylated 40 nm polystyrene (PS) NPs. The loading was shown to rely on energy-dependent cellular processes. The aim of the current study is to investigate the possible role of the membrane-associated glycans and NP size on the transient PS NP loading of HPCs. To this end, HPCs were exposed to 40, 100 and 200 nm PS NPs which are chemically identical to the 40 nm PS NPs as used in our previous work [26]. We furthermore studied the loading behavior after cells were treated with the proteoglycan-degrading enzyme neuraminidase. The human peripheral blood-derived THP-1 monocyte cell line, with known NP phagocytosis capacity [27, 28] was investigated in parallel to evaluate the cell-specific character of the NP loading behavior. Flow cytometry and confocal imaging were applied on live HPCs and THP-1 cells to evaluate their NP uptake behavior.

## Results

### Nanoparticle characterization

Prior to cell exposure experiments, stock suspensions of PS NPs were subjected to quality control to confirm their sizes and stability. Size distribution of the NPs was evaluated by means of nanoparticle tracking analysis (NTA) and differential centrifugal sedimentation (DCS), measuring the hydrodynamic particle diameter (Additional file 1: Table S1). In addition, transmission electron microscopy (TEM) analysis of the PS NPs was performed, revealing the mean primary diameters of 42, 100 and 230 nm, respectively (Additional file 1: Table S1, Fig. S1). As expected, hydrodynamic diameters observed by NTA and DCS were slightly larger (respectively, 55, 125 and 260 nm for NTA, and 63, 110 and 245 nm for DCS). For all three PS NP sizes, good colloidal stability was demonstrated by both TEM and zeta potential analysis (Additional file 1: Table S1). Furthermore, zeta potential was used as an indirect measure to estimate the density of surface carboxyl groups on the different PS NPs, as described by Zhu et al. [29] (Additional file 1: Table S1). Carboxylic acid densities were observed to be high, as indicated by the manufacturer, and to increase with increasing size. Characterization of the PS NPs after incubation in cell culture medium resulted in an increase of diameter and an increase of the zeta potential to a similar value of − 23 mV for the different particles, indicating adsorption of serum-derived biomolecules to the NP’ surfaces (Additional file 1: Table S2, Fig. S2). The colloidal stability of the particles was not impaired by this shell of biomolecules.

### Differential NP loading behavior of HPCs and THP-1 cells

As described previously [26], CD34+ HPCs were demonstrated to associate in a transient manner with 40 nm PS NPs in contrast to dendritic cells. Cell-associated fluorescence intensity (FI), originating upon exposure to PS NPs, reached a maximum after ~ 1 h and declined afterwards, suggesting prompt accumulation of NPs by the cells followed by NP release. To study whether this observation was dependent on NP size, we exposed HPCs to 50 µg/ml of 40, 100 and 200 nm PS NPs. The latter concentration was selected based on our previous work [26], in which it was demonstrated to generate a potent loading capacity of HPCs with 40 nm PS NPs in the linear part of a dose–response curve after 24 h of exposure. In parallel, we performed a similar experiment with THP-1 monocytes to compare the cell-specific time course of the association between PS NPs of different size and cells.

**Fig. 1:**
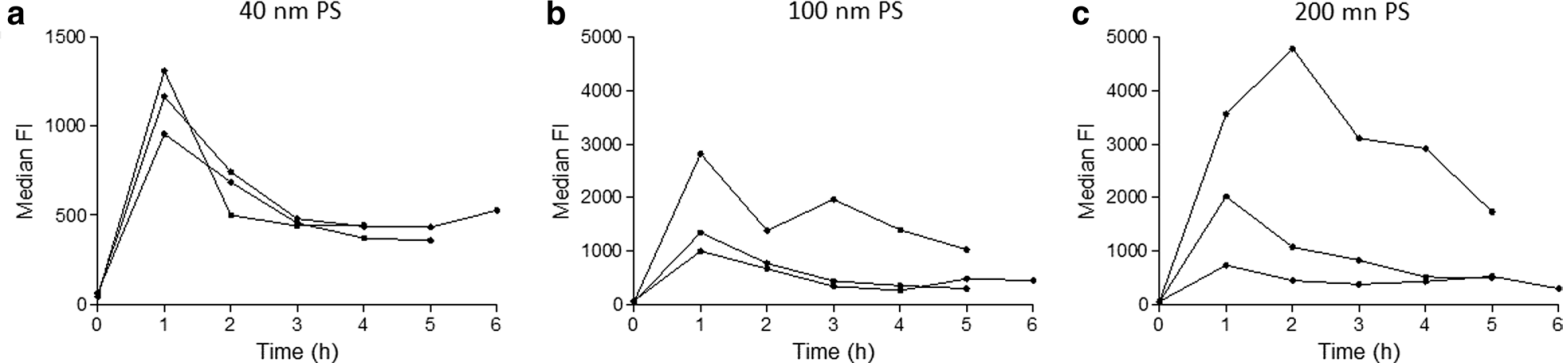
Transient loading kinetics of CD34^+^ HPCs demonstrated as median fluorescence intensity (FI) obtained by flow cytometry. CD34^+^ HPC cultures were exposed to 50 µg/ml of 40 (**a**), 100 (**b**) or 200 nm (**c**) PS NPs and measured up to 6 h. Data of HPC cultures obtained from 3 different cord blood samples are shown

**Fig. 2:**
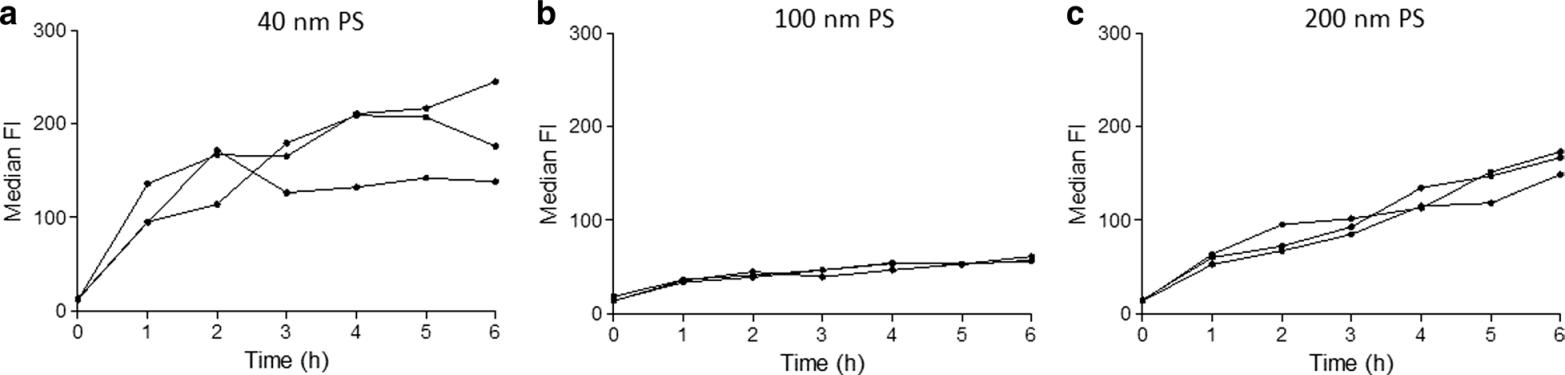
Time-evolution of the loading of THP-1 cells demonstrated as median fluorescence intensity (FI) obtained by flow cytometry. THP-1 cells were exposed to 50 µg/ml of 40 (**a**), 100 (**b**) or 200 nm (**c**) PS NPs and monitored up to 6 h. Profiles of 3 THP-1 cultures with different passage number are shown

Flow cytometry was used to evaluate early NP loading of the cells up to 6 h. We were able to confirm the transient loading behavior of HPCs using 40 nm PS NPs (Fig. 1a). A similar transient loading was observed for 100 and 200 nm PS NPs, although in this case the number of NPs in contact with the cells was approximately 25 and 160-fold lower compared to 40 nm PS NPs, respectively. The median FI reached a maximum after ~ 1 h, after which the signal declined (Fig. 1b, c). For the same NP size fluorescence signals varied between different HPC donors. In all cases, the cell-associated fluorescence signal diminished to stable, low values, suggesting NP release to a minimal level. In case of the THP-1 cells, a persistent increase of the cell-associated fluorescence was observed, indicating monotonous time-dependent association kinetics (Fig. 2). Thus, the time-dependence of the association is substantially different between HPCs and THP-1 cells.

### Extracellular retention of NPs and influences of membrane-associated proteoglycans

**Fig. 3:**
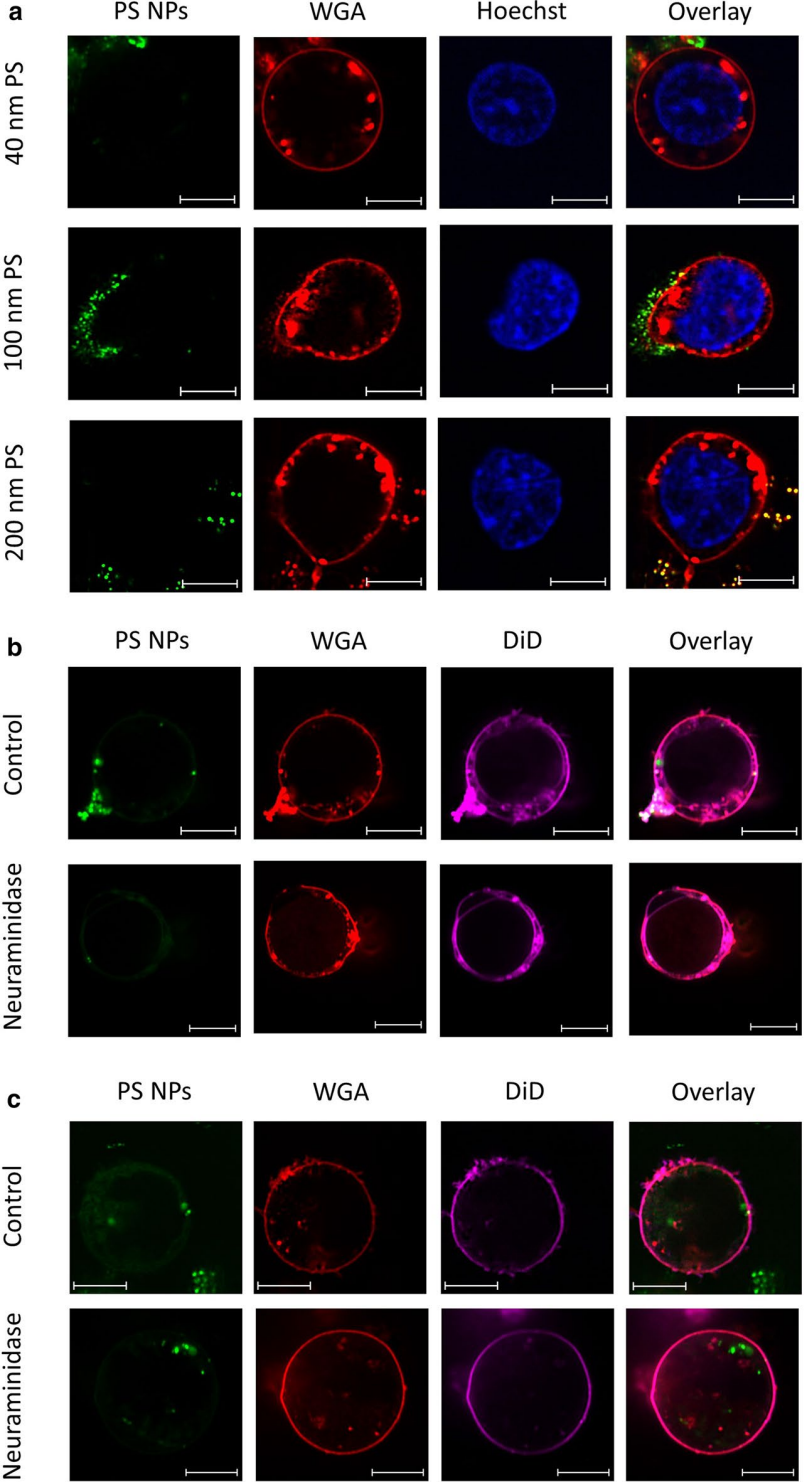
Confocal images of HPCs exposed to 50 µg/ml of PS NPs. **a** Demonstrates HPCs exposed to PS NPs of 40 nm, 100 nm and 200 nm for 1 h. **b**, **c** Display HPCs exposed to 50 µg/ml of 40 nm PS NPs for 1 h and 4 h, respectively, and whether or not (‘control’) treated with 0.83 U/ml neuraminidase for 30 min prior to PS NP exposure. PS NPs are yellow-green fluorescently labelled (green) (**a**–**c**), cell membrane-associated proteoglycans were stained by Alexa Fluor^®^ 555 conjugated wheat germ agglutinin (red) (**a**–**c**), nuclei were stained using Hoechst 33342 (blue) (**a**) and the lipid bilayer was stained using DiD (violet) (**b**, **c**). Images were collected between 30 min and 1 h after loading and subsequent staining. Scale bar: 5 µm

**Fig. 4:**
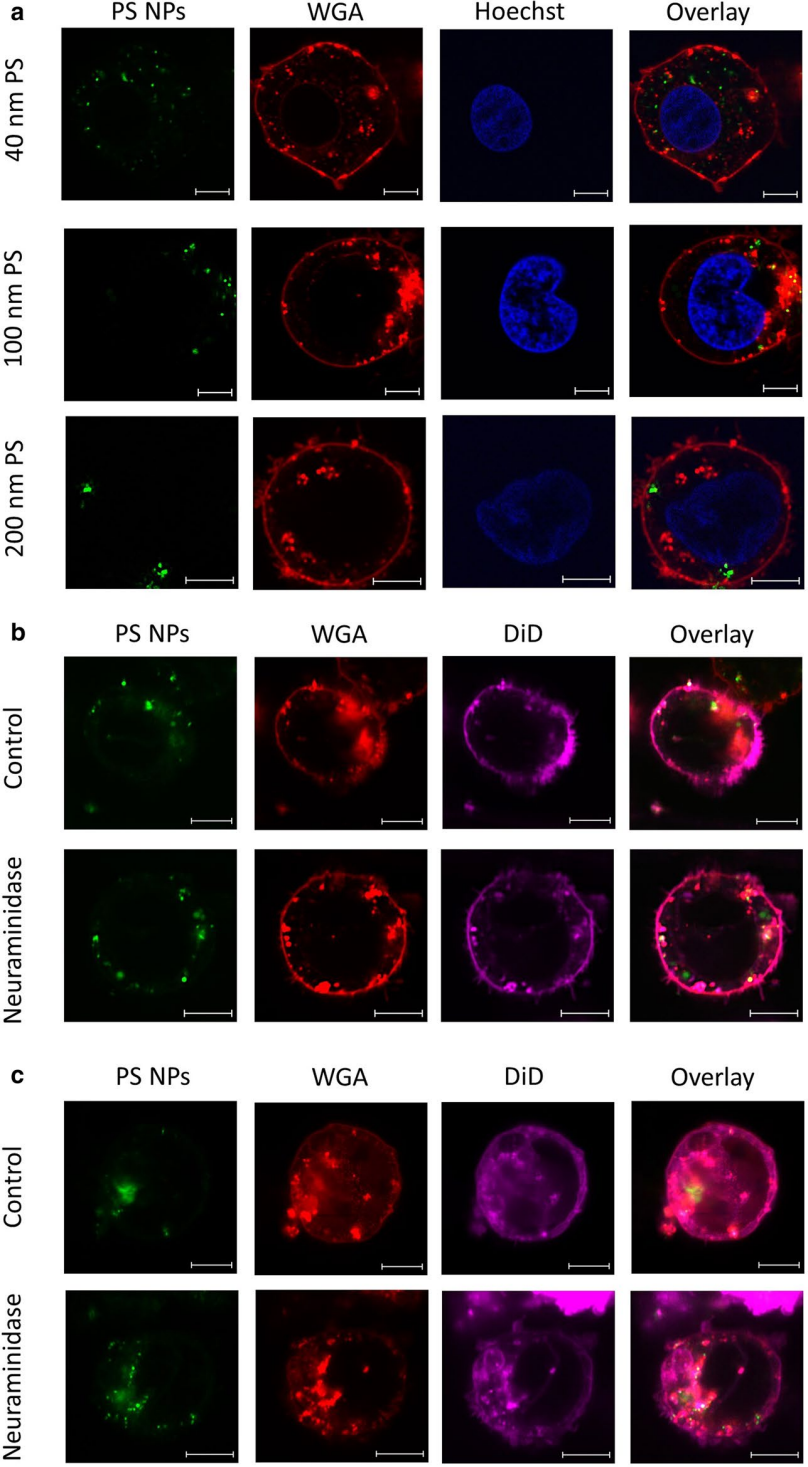
Confocal images of THP-1 cells exposed to 50 µg/ml of PS NPs. **a** Demonstrates THP-1 cells exposed to PS NPs of 40 nm, 100 nm and 200 nm for 4 h. **b**, **c** Display THP-1 cells exposed to 50 µg/ml of 40 nm PS NPs for 1 h and 4 h, respectively, and whether or not (‘Control’) treated with 0.83 U/ml neuraminidase for 30 min prior to PS NP exposure. PS NPs are yellow-green fluorescently labelled (green) (**a**–**c**), cell membrane associated proteoglycans were stained by Alexa Fluor^®^ 555 conjugated wheat germ agglutinin (red) (**a**–**c**), nuclei were stained using Hoechst 33342 (blue) (**a**) and the lipid bilayer was stained using DiD (violet) (**b**, **c**). Images were collected between 30 min and 1 h after loading and subsequent staining. Scale bar: 5 µm

Confocal microscopy was used to evaluate the localization of the PS NPs in relation to the cell membrane (outside and/or inside) for HPCs (Fig. 3) and THP-1 cells (Fig. 4). Both cell types were incubated for 1 h and 4 h with 40 nm PS NPs. These incubation times were applied to obtain optimal fluorescent intensities associated with the two cell types (1 h for HPCs, 4 h for THP-1), and were based on the flow cytometry data. HPCs showed limited numbers of internalized NPs after 1 h, corresponding to the time of peak loading by these cells. More specifically, despite applying 3 washing steps prior to analysis, NPs appeared adsorbed in small clusters at the outer membrane and some NPs coincided with WGA-stained structures, but quasi no particles were observed inside the cells [Fig. 3a (upper panel) and Fig. 3b (control panel)]. The same behaviour was observed for the 3 different PS NP sizes (Fig. 3a). However, over time this association changed, as demonstrated for the 40 nm PS NPs, which were also found in the intracellular environment after 4 h (Fig. 3c, control panel). In contrast, after 1 h, THP-1 cells demonstrated clear internalization of 40 nm PS NPs, but no accumulation at the outer membrane (Fig. 4b, control panel). In these cells, increased numbers of 40 nm PS NPs, as well as accumulation of 100 and 200 nm PS NPs were found in the intracellular compartment after incubation for 4 h [Fig. 4a, c (control panel)]. In both cell types, part of the internalized NPs appear at the same location as cellular structures stained by WGA.

**Fig. 5:**
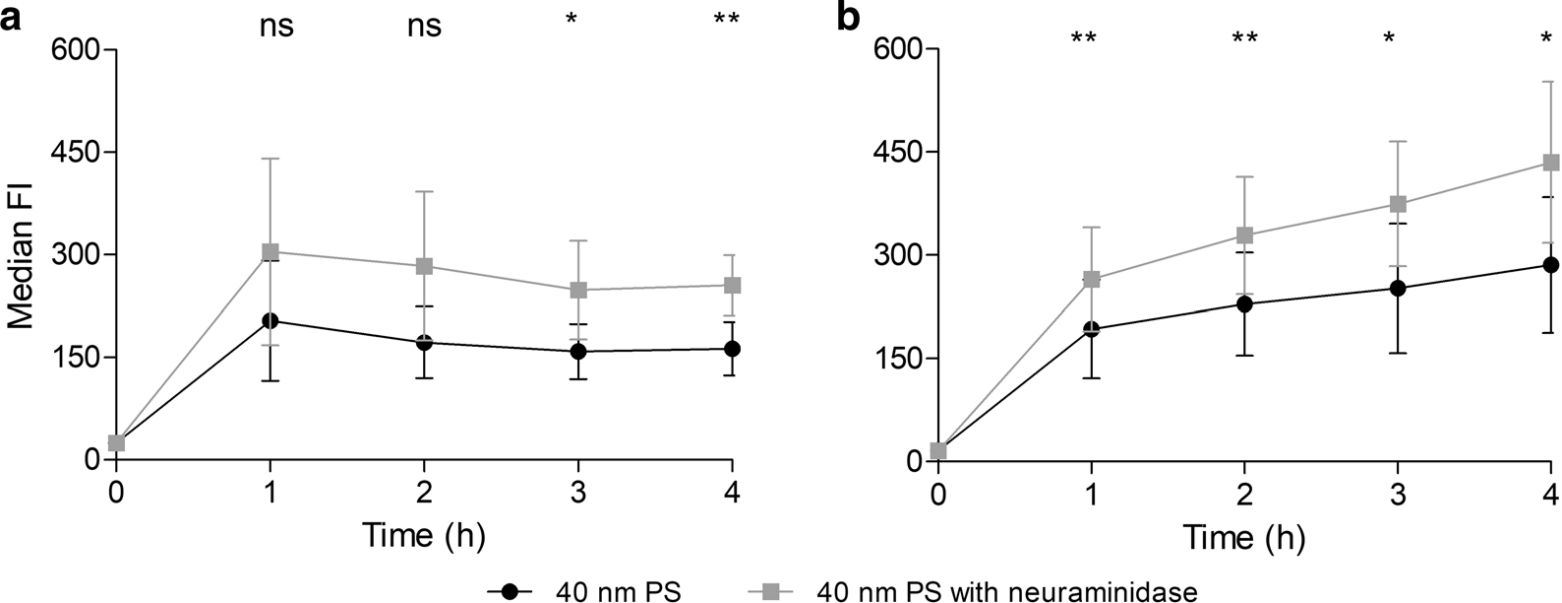
Time-evolution of PS NP loading by HPCs (**a**) and THP-1 (**b**) cells demonstrated as median fluorescence intensity obtained by flow cytometry. Both cell types were pre-incubated with 0.83 U/ml neuraminidase before exposure to 50 µg/ml of 40 nm PS NPs, and were compared to cells only exposed to PS NPs. Mean and standard deviation of HPC cultures obtained from 3 different cord blood samples (**a**) and of 3 THP-1 cultures with different passage number are shown (**b**). Two-tailed, paired t-test: *p-value < 0.05; **p-value < 0.01

Given the observed temporary retention of PS NPs at the HPCs’ outer plasma membrane, we tested the possible involvement of membrane-associated proteoglycans in this process, as has been described for endothelial glycocalyx [23, 24]. Firstly, using ruthenium red staining and ultrastructural TEM analysis of HPCs (Additional file 1: Fig. S3), we confirmed the presence of membrane-associated proteoglycan granules [30]. Next, HPCs and THP-1 cells were treated with the sialic acid-hydrolyzing neuraminidase for 30 min, prior to time-dependent exposure experiments with 40 nm PS NPs. Cell-specific time courses of NP association in HPCs and THP-1 cells remained persistent after cells were enzymatically treated (Fig. 5). However, a statistically significant increase in median FI could be observed between treated and untreated cells from 3 h exposure onwards for the HPCs and from 1 h for the THP-1 cells, suggesting an effect of the enzyme under the given experimental circumstances. Moreover, when investigating the time-dependent effect of cleavage of sialic acid residues on PS NP localization by means of confocal microscopy (Figs. 3b, c and 4b, c), in both cell types a clearly increased internalization of NPs could be observed after 4 h NP exposure in treated (Neuraminidase) versus untreated (control) cells (Figs. 3c and 4c).

### Cytotoxicity assessment of PS NPs and neuraminidase

By using the PrestoBlue™ cell viability reagent, potential cytotoxic effects of both PS NPs and the enzyme neuraminidase on the THP-1 cells were studied (Additional file 1: Fig. S4). No adverse health effects were observed for all three sizes of PS NPs, which is in line with our previous observation of absence of cytotoxicity of the 40 nm PS NPs in HPCs after 24 h of exposure [26]. Moreover, co-exposure of these NPs with neuraminidase did not result in decreased cell viability. Taken together, these data demonstrate that the selected concentrations of both the PS NPs and neuraminidase were safe to use in the cell exposure experiments.

## Discussion

The focus of this study was to investigate whether NP size or membrane-associated glycans can influence the time-evolution of association of NPs in HPCs and compare this with the behavior of THP-1 monocytes. In previous work [26], we demonstrated that 40 nm carboxylated PS NPs associate in high numbers with HPCs until 1 h after exposure, upon which the major proportion tend to dissociate from the cells. By cooling down the cells to 4 °C, it was proven that the transient effect was based on energy-dependent processes [26].

In the current work, we extended the observations of the 40 nm PS NPs by comparing the handling of nanoparticles by naive HPCs to THP-1 monocytes which represent a mature type of suspension cells with established phagocytosing capacity. Major differences in NP loading course could be observed between the THP-1 cells and the HPCs upon continuous exposure to PS NPs. The HPCs promptly accumulate high numbers of NPs followed by a decline in loading, while in case of THP-1 cells, the uptake is started immediately, followed by a monotonously increasing pattern. In contrast to the THP-1 cells, HPCs clearly show accumulation of NPs at the outer cellular membrane at their peak loading 1 h post exposure. Over time, this association changes and NPs start to appear at the interior of the HPCs. Our findings complement those of Brüstle et al. [31], who described that 120 nm carboxylated PS NPs were internalized by hematopoietic stem cells after 24 h of exposure, whereas we demonstrated internalization after 4 h of exposure. The group of Brüstle also applied confocal microscopy to evaluate NP internalization, but used different experimental conditions. In their study, cells were exposed to 300 µg/ml PS NPs, which is a sixfold higher dose than applied in the current study. Also, Brüstle et al. [31] obtained hematopoietic cells from peripheral blood, whereas umbilical cord blood was used in the present study. The cells of the hematopoietic system have been studied intensively in the past. Differences between cord blood stem cells and their counterparts in the peripheral blood or bone marrow have been described and have to be considered when studying uptake behavior [32].

As described in literature, there is an optimal size for NP adsorption and internalization, which is specific for each cell type [33, 34]. Moreover, this optimal size will change upon different surface functionalities. Therefore, besides the 40 nm PS NPs HPCs and THP-1 cells were exposed to PS NPs with a distinct diameter of 100 and 200 nm. The NP surfaces were functionalized with carboxyl groups, showing higher carboxyl acid density with increasing size. The differences between the corresponding zeta potentials of the PS NPs suspended in water, however, disappeared when the different NPs were incubated in serum-supplemented culture medium under cell exposure conditions. This is most probably due to the adsorption of biomolecules on the NPs’ surfaces, which we have observed for the 40 nm PS NPs to occur instantly [26]. Therefore, it is reasonable to conclude that the differences in carboxylation density of the studied PS NPs do not have a major impact on the observed time profiles of NP internalization.

The three different NP sizes all presented a similar cell-specific time course of association with the HPCs and THP-1 cells, although median FI peak values varied between different PS NPs’ sizes. The latter is not surprising since we applied equal mass concentrations of the different PS NPs to the cells, representing different numbers of particles, and each size shows different numbers of embedded fluorescent molecules per NP (Additional file 1: Table S1). Yet, our data suggest that the transient time course of PS NP association with HPC is insensitive to NP size. This observation is at variance with the results of Varela et al. [35]. In the latter publication, 40 nm PS NPs were demonstrated to be internalized faster than 20 or 100 nm PS NPs by both 1321N1 human astrocytoma and A549 human lung carcinoma cell lines [36]. In the case of the currently investigated THP-1 cells, changing the particle diameter of PS NPs altered the slope of the uptake kinetics, also suggesting an effect of NP size on their association with the cells. Indeed, 40 nm NPs tended to be internalized more rapidly by THP-1 cells than 100 and 200 nm PS NPs, which is in correspondence with the study of Varela et al. [35]. This phenomenon was also demonstrated in other studies, where different sizes of gold NPs were demonstrated to be internalized by different processes in varying cell types [34, 37]. Another key factor in NP internalization behaviour by cells, affecting mainly the initial membrane adsorption, is the transport mechanism of NPs suspended in the surrounding fluid. In general, the diffusion coefficient of NPs is inversely correlated with their size, and for larger particles or aggregates transport may be driven by sedimentation. In this study, however, no obvious sign of NP sedimentation was noticed and the colloidal stability of the three PS NPs under experimental cell culture conditions was confirmed. Finally, since equal mass concentrations were applied here for the different sizes of NPs, the cells were exposed to different particle numbers, which also may affect the cellular interaction and internalization process. Despite the above mentioned variables relating to NP properties and dosimetry in our experimental set-up and given the fact that equal cell numbers of both cell types were exposed to the NP suspensions, this study highlights that the transient PS NP loading capacity of HPCs that is observed at short time intervals is consistent and rather unique compared to phagocytic cells (THP-1 in this study, and dendritic cells in Deville et al. [26]).

Besides NP properties, cell characteristics can influence the fate of NPs upon cellular interaction. Structures and molecules such as proteoglycans at the outer surface can influence the uptake of NPs by cells [38]. Multiple studies have already demonstrated that the glycocalyx has a regulating role in the handling of NPs [23, 24, 38]. For example, it has been shown that uptake of PS NPs by endothelial cells was impeded by the glycocalyx of the cells. In our current study, WGA was used to visualize the proteoglycans at the outer membrane of the HPCs and THP-1 cells because it is known to recognize and bind sialic acid and *N*-acetylglucosamine residues [38]. Our confocal images of HPCs at peak loading time (1 h post-exposure) demonstrate that the PS NPs appeared in clusters in well-defined WGA-positive regions closely associated with, but separated from the outer plasma membrane, as if they were entrapped in carbohydrate structures (Fig. 3). A few single NPs appeared closely attached to the cell membrane, but quasi no particles were observed inside the cells. To assess the potential role of proteoglycans in the observed extracellular NP retention, changes in the membrane-associated proteoglycans were induced by treatment with neuraminidase, a sialic acid hydrolase cleaving terminal residues of the glycocalyx, thus reducing the interaction with WGA. Using a double-staining protocol with WGA and DiD in confocal imaging, both the membrane-associated proteoglycans and the intact lipid bilayer could be visualized simultaneously with the NP association. Based on flow cytometry measurements and the confocal images after neuraminidase treatment, respectively, we observed a consistent increase of cell-associated PS NPs’ fluorescence over time, and an increased amount of internalized NPs after 4 h exposure in HPCs and THP-1 monocytes. Despite the increased median FI over time, the overall time course of the loading behavior remained unchanged. This suggests that by removing the terminal sialic acid-rich regions, and thereby their contribution to a negative charge on the cells’ surfaces, the membrane becomes more accessible to NP association.

Taken together, our data for the first time demonstrate the involvement of membrane-associated proteoglycans, such as sialic acids, in the membrane interaction of NPs with HPCs and THP-1 cells. It should be noted, however, that the interaction between NPs and proteoglycans is not limited to sialic acids.

## Conclusion

In this study it was demonstrated that upon in vitro exposure HPCs initially accumulate PS NPs at their outer membrane followed by an intracellular association over time. NP size did not affect this transient loading behavior of PS NPs in HPCs. In contrast, THP-1 cells clearly showed internalization of the PS NPs at all observed time points, with differences sensitive to NP size. Cell surface glycans were found to contribute to the retention of PS NPs in both cell types. Thus, we revealed the prolonged initial NP retention at the outer cell membrane to be a unique characteristic of the HPCs, which is promising to be applied in the intelligent design of NP-based applications in HPC-based regenerative protocols.

## Materials and methods

### Nanoparticle characterization

Yellow-green (λ^Ex^ = 505 nm, λ^Em^ = 515 nm) carboxylated PS NPs of 40, 100 and 200 nm were purchased from Invitrogen (Thermo Scientific, Waltham, Massachusetts, USA). The NP batches were characterized for NP size by means of nanoparticle tracking analysis (NTA) using an NS500 instrument (NanoSight Ltd, Wiltshire, UK) and, by differential centrifugal sedimentation (DCS) using a disc centrifuge DC24000 (CPS Instruments, Prairieville, LA, USA) and for zeta potential using ZetaPALS (Brookhaven Instruments Corporation, Holtsville, NY, USA). For characterization, the NP stock solutions were diluted in ultrapure water and immediately measured, or dispersed in cell culture medium containing 10% foetal bovine serum (FBS Superior; PAA Laboratories, Pasching, Austria) and incubated for 4 h at 37 °C following a similar protocol as used for the cell exposure experiments (vide infra). The NP dispersions for zeta potential analysis were prepared in 1 mM KCl before measurement, medium-incubated NPs were first collected by centrifugation at 25,000×*g* up to 1 h. Fluorescence intensity per particle was determined based on fluorescence measurements of NP samples diluted in well-defined volumes of ultrapure water using a Clariostar microplate reader (BMG Labtech, Ortenberg, Germany) and particle concentrations obtained from the manufacturer.

### Isolation and culture of CD34^+^ HPCs

Procedures for isolation and suspension culture of CD34+ HPCs have been described previously [26]. Briefly, human cord blood samples were collected from umbilical cord blood vessels of placentas of full-term infants, born at the Heilig Hart Hospital in Mol and Sint-Dimpna Hospital in Geel, Belgium. Informed consent was given by the mothers and the study was approved by the ethical committee of the University Hospital of Antwerp and University of Antwerp. Mononuclear cells were separated from human cord blood samples by density gradient centrifugation (Ficoll-Paque™ Plus, GE Healthcare, Uppsala, Sweden) and stored overnight at 4 °C. On the next day, CD34+ HPCs were extracted using a positive immunomagnetic selection method (EasySep^®^ Human CD34 Positive Selection Kit I, Stem cell Technologies, Grenoble, France) according to the manufacturer’s guidelines. The viability (91% ± 4% [n = 6]) of the purified HPCs was determined by counting cell numbers using an automatic cell counting device Nucleocounter (Chemometec, Allerod, Denmark). Immediately after isolation, the HPCs were applied in NP experiments and they were maintained in Iscove’s Modified Dulbecco’s Medium (IMDM; Gibco, Paisley, UK) supplemented with 10% FBS (FBS Superior; PAA Laboratories), 2% penicillin/streptomycin (P/S; 5000 U/ml–5000 μg/ml; Gibco) and 1% bovine serum albumin (BSA; Sigma-Aldrich Co., St Louis, Missouri, USA).

### THP-1 cell culture

The human monocytic THP-1 cell line was obtained from American Type Culture Collection (ATCC; Manassas, Virginia, USA). They were maintained in suspension in RPMI 1640 medium supplemented with 10% FBS (FBS Superior; PAA Laboratories) at 37 °C in a humidified incubator with 5% CO_2_. THP-1 cells were used from passage 5 to 25. Subculturing was performed every 3 days by adding fresh culture medium to obtain a dilution of 2–4 × 10^5^ viable cells/ml.

### Nanoparticle dispersion and cell exposure

Prior to NP exposure, NP stock suspensions were vortexed for 30 s and were dispersed by adding them to pre-warmed cell culture medium (37 °C) to obtain a concentration of 0.5 mg/ml. Next, these NP dispersions were added to the cell suspensions by gentle pipetting in a ratio of 1:10 (v/v) to obtain a final NP concentration of 50 µg/ml and cell density of 10^6^ cells/ml. Lastly, the NP-cell suspension was seeded in 96-well plates at 100 µl per well, and incubated at 37 °C and 5% CO_2_ until analysis. For the enzymatic degradation of the membrane-associated sialic acids prior to NP exposure of the cells, cells were incubated in cell culture medium containing 0.83 U/ml neuraminidase from *Clostridium perfringens* (Sigma Aldrich) for 30 min. Afterwards, cells were pelleted and resuspended in fresh pre-warmed culture medium (37 °C) to which NP dispersions were added. Control cells were treated only with cell culture medium.

### Assessment of cytotoxicity of PS NPs and neuraminidase

Potential cytotoxic effects of the PS NPs and the enzyme neuraminidase were evaluated using the PrestoBlue™ Cell viability reagent (Invitrogen). THP-1 cells were incubated with 0.83 U/ml neuraminidase, 50 µg/ml of PS NPs, or with both as described above at 37 °C and 5% CO_2_ for 1 or 4 h. Next, cells were collected by centrifugation to remove the NPs, and resuspended in fresh cell culture medium containing the PrestoBlue™ cell viability reagent at 10% (v/v). After incubation at 37 °C and 5% CO_2_ for 2 h, fluorescence intensities were analyzed using a Clariostar microplate reader (BMG Labtech, Ortenberg, Germany) in the top-optic mode, with the excitation wavelength set at 552 (bandwidth 20 nm) nm and the emission wavelength detected at 599 nm (bandwidth 22 nm). Staurosporine (50 and 10 µM for 1 and 4 h cell exposure time, respectively) was used as a control condition for impaired cell viability.

### Flow cytometry

NP loading of the HPCs and THP-1 cells was evaluated using flow cytometry (FACS Calibur™, Becton–Dickinson, San Jose, CA, USA). Hereto, quality aspects regarding cell culture, NP dispersions, cellular exposure, flow cytometric measurements and analyses of results, as described by Salvati et al. [39], were taken into account. After fixed incubation times of 0, 1, 2, 3, 4, 5 and 6 h, NP-exposed cells were transferred to polystyrene tubes (Becton–Dickinson) and washed three times with phosphate buffer saline (PBS, Gibco, Paisley, UK) without Ca^2+^ and Mg^2+^ to remove non-associated NPs and collected by centrifugation. Viable HPCs and THP-1 cells were identified by light scattering using gates to exclude dead cells and cell debris. For each sample, sample collection was terminated when 10.000 events in the gated region were acquired or after 3 min of collection. Light scattering profiles and fluorescence histograms were evaluated using CellQuest™ software, version 6 (Becton–Dickinson). Kaluza^®^ Flow Analysis software (Beckman coulter, Brea, CA, USA) was used to display flow cytometry plots and histograms for publication. For data analysis, NP-loaded cells were distinguished by applying a marker to the autofluorescence histograms of untreated cells (Additional file 1: Fig. S5, Fig. S6). Hence, all cells with a fluorescence signal above the marker limit are assumed to be NP-loaded. NP loading was evaluated by means of the median value of the fluorescence intensity (Median FI) as a robust measure for the central tendency of possibly skewed probability distributions. Different photomultiplier voltages and compensation settings were applied for HPCs and THP-1 cells.

Paired, two-tailed t-tests were performed using GraphPad Prism version 5.00 for Windows, GraphPad Software (La Jolla California USA).

### Confocal microscopy

After the non-adherent HPCs and THP-1 cells were exposed to 50 μg/ml of PS NPs for 1 (HPCs) and 4 h (THP-1), cells were washed three times with PBS, and were labelled with 5 μg/ml Alexa Fluor^®^ 555-conjugated wheat germ agglutinin (WGA; Invitrogen) at 37 °C for 15 min. Next, cells were washed by centrifugation and adding fresh pre-warmed PBS (37 °C). After washing, cell nuclei were stained by adding Hoechst 33342 (NucBlue^®^ Live ReadyProbes^®^, Invitrogen) and live cells were transferred to a cytocapture chamber (GE Healthcare) and incubated for 15 min at 37 °C to allow the cells to settle down. Visualization of the cellular proteoglycans was performed by a co-staining of both glycan structures and the lipid bilayer by Alexa Fluor^®^ 555-conjugated WGA and 1,1-dioctadecyl-3,3,3,3-tetramethylindodicarbocyanine (DiD; Invitrogen) respectively. Confocal imaging with improved spatial resolution was performed using a LSM 880 Airyscan installed on an inverted Axio Observer (Carl Zeiss, Jena, Germany) and equipped with a 63x/1.4 NA oil immersion objective (Carl Zeiss, Jena, Germany). Pixel size varied between 43 nm and 48 nm, measured with dwell times between 1.85 µs and 15.9 µs per pixel. Pinholes were set at 128 µm for PS NPs and Alexa Fluor 555 channels for both cell types. For the Hoechst 33342 channel, pinholes were set at 76 µm for HPCs and at 38 µm for THP-1 cells. Z-stacks were recorded with a 180 nm interval, spanning a range of 9 µm to 13 µm to image each cell from top to bottom, with an axial resolution of 0.4 µm. The different colors present in these cells were recorded using sequential frames. Alexa fluor^®^ 555-labelled WGA and yellow-green PS NPs were excited by using respectively a 543 nm helium–neon laser of 0.64 mW and a 488 nm line of 25 mW air-cooled argon-ion laser, both under the control of an acousto-optical tunable filter. The lipid dye DiD was excited by using a 5 mW 633 nm He–Ne laser. For WGA, DiD and PS NPs, the emission light was detected using an Airyscan detector, after passing a main dichroic (MBS488/543) and an emission filter (LP 525 and BP555-620 for WGA, BP495-550 for PS NPs). In addition, Hoechst 33342 was excited by means of a 120 fs pulsed laser light of a Titanium:Sapphire laser (MaiTai DeepSee, Spectra-Physics, Santa Clara, USA) tuned at an output wavelength of 730 nm. An MBS 690 was used as main dichroic mirror and the spectral Quasar detector (from 409 nm to 473 nm) was used to detect the emission light. ZEN Black software version 14 (Carl Zeiss, Jena, Germany) was used to process the data from the Airyscan detector using a Zeiss proprietary algorithm.

## Additional file

**Additional file 1.** The additional data includes information regarding the TEM protocol, TEM images of the PS NPs and HPCs, data on NP characterization and cell viability assessment. Furthermore, flow cytometry gating strategies are included.

## Data Availability

All data generated or analysed during this study are included in this published article and its Additional files.

## Abbreviations

A.U.: arbitrary unit
BSA: bovine serum albumin
DCS: differential centrifugal sedimentation
DiD: 1,1-dioctadecyl-3,3,3,3-tetramethylindodicarbocyanine
FBS: foetal bovine serum
FI: fluorescence intensity
GAG: glycosaminoglycan
HPC: hematopoietic progenitor cell
IMDM: Iscove’s Modified Dulbecco’s Medium
NP: nanoparticle
NTA: nanoparticle tracking analysis
PBS: phosphate buffer saline
PS: polystyrene
SD: standard deviation
TEM: transmission electron microscopy
WGA: wheat germ agglutinin
