# Supplementary material for: Role of nanoparticle size and sialic acids in the distinct time-evolution profiles of nanoparticle uptake in hematopoietic progenitor cells and monocytes
Source: J Nanobiotechnology. 2019 May 13;17:62. doi: 10.1186/s12951-019-0495-x (PMC6513515; doi:10.1186/s12951-019-0495-x)
Supplement: Supplementary file 1 — Additional file 1. The additional data includes information regarding the TEM protocol, TEM images of the PS NPs and HPCs, data on NP characterization and cell viability assessment. Furthermore, flow cytometry gating strategies are included. [file 12951_2019_495_MOESM1_ESM.docx]

# Additional information

**Role of nanoparticle size and sialic acids in the distinct time-evolution profiles of nanoparticle uptake in hematopoietic progenitor cells and monocytes**

Bart Wathiong^1^, Sarah Deville^1^, An Jacobs^1^, Nick Smisdom^2^, Pascal Gervois^2^, Ivo Lambrichts^2^, Marcel Ameloot^2^, Jef Hooyberghs^1,3^, Inge Nelissen^1^

^1^Health Department, Flemish Institute For Technological Research (VITO), Boeretang 200, B-2400 Mol, Belgium

^2^Biomedical Research Institute (BIOMED), Hasselt University, Agoralaan building C, B-3590 Diepenbeek, Belgium.

^3^Theoretical physics, Hasselt University, Agoralaan building D, B-3590 Diepenbeek, Belgium

## Transmission electron microscopy

An aqueous (ultrapure water) solution of 40 nm, 100 nm or 200 nm nanoparticles (5 µg/ml) was dispersed and dried on 0.7% formvar-coated copper grids. TEM analysis was performed with a Philips EM208 S electron microscope operated at 80 kV (Philips, Eindhoven, The Netherlands) and equipped with a Morada Soft Imaging System and compatible iTEM-FEI software (Olympus SIS, Münster, Germany). To determine nanoparticle size, at least 185 particles of each size category were manually measured using the metric plugin of the iTEM-FEI software package (Figure S1).

For analysis of the membrane-associated proteoglycans (Figure S3), THP-1 and HPC samples were pelleted at 300 g for 5 min after which they were fixed in 2% glutaraldehyde with 0.15% ruthenium red. Post-fixation was performed with 2% osmium tetroxide and 0.15% ruthenium red in 0.05 M sodium cacodylate buffer for 1 hour at 4°C. Dehydration of the samples was performed by ascending concentrations of aceton and the dehydrated samples were impregnated overnight in a 1:1 mixture of aceton and araldite. The impregnated samples were embedded in araldite at 60°C and were cut in slices of 70 nm with a Leica EM UC6 microtome (Leica, Wetzal, Germany) and transferred to 0.7% formvar-coated copper grids. The samples were contrasted with 0.5% uranyl acetate and lead citrate using a Leica EM AC20 (Leica). Analysis was performed as described above.

**Table S1:** Characterization of PS NPs stock suspensions in aqueous dispersions.

|  |  | **40 nm PS** | **100 nm PS** | **200 nm PS** |
| --- | --- | --- | --- | --- |
| NTA | Mean diameter ± SD (nm) | 55.4 ± 0.3 | 125.2 ± 0.5 | 260 ± 20 |
| TEM | Mean diameter ± SD (nm) | 41.8 ± 9.7 | 99.6 ± 9.9 | 230 ± 40 |
| DCS | Peak diameter (nm) | 62.6 | 110.5 | 245.2 |
| Zeta potential | Mean ± SD (mV) | -42 ± 2 | -51 ± 3 | -60 ± 2 |
| Particle carboxylation (carboxyl acids/nm²)^(*)^ | | 1.5 | 1.7 | 2.0 |
| Particle concentration (particles/ml) ^(**)^ | | 1.0 x 10^15^ | 3.8 x 10^13^ | 6.2 x 10^12^ |
| Fluorescence intensity per NP (A.U.) | | 2.3 x 10^-7^ | 4.0 x 10^-6^ | 2.8 x 10^-5^ |

Fluorescent intensity per NP was calculated based on experimentally determined intensities. Number concentrations were provided by the manufacturer.

^(*)^ Carboxylation density calculated based on the zeta potential as described by Zhu et al. (2013)

^(**)^ Concentration obtained from manufacturer

**Abbreviations:** SD, standard deviation; A.U., arbitrary unit

**Table S2:** Characterization of PS NP dispersions in cell culture medium.

|  |  | **40 nm PS** | **100 nm PS** | **200 nm PS** |
| --- | --- | --- | --- | --- |
| NTA | Mean diameter ± SD (nm) | 66.2 ± 1.5 | 142 ± 3 | 294.8 ± 8.9 |
| DCS | Peak diameter (nm) | 86.2 | 143.6 | 259.2 |
| Zeta potential | Mean ± SD (mV) | -22.68 ± 1.11 | -23.78 ± 0.6 | -23.17 ± 0.58 |

**Abbreviations:** SD, standard deviation; A.U., arbitrary unit

**
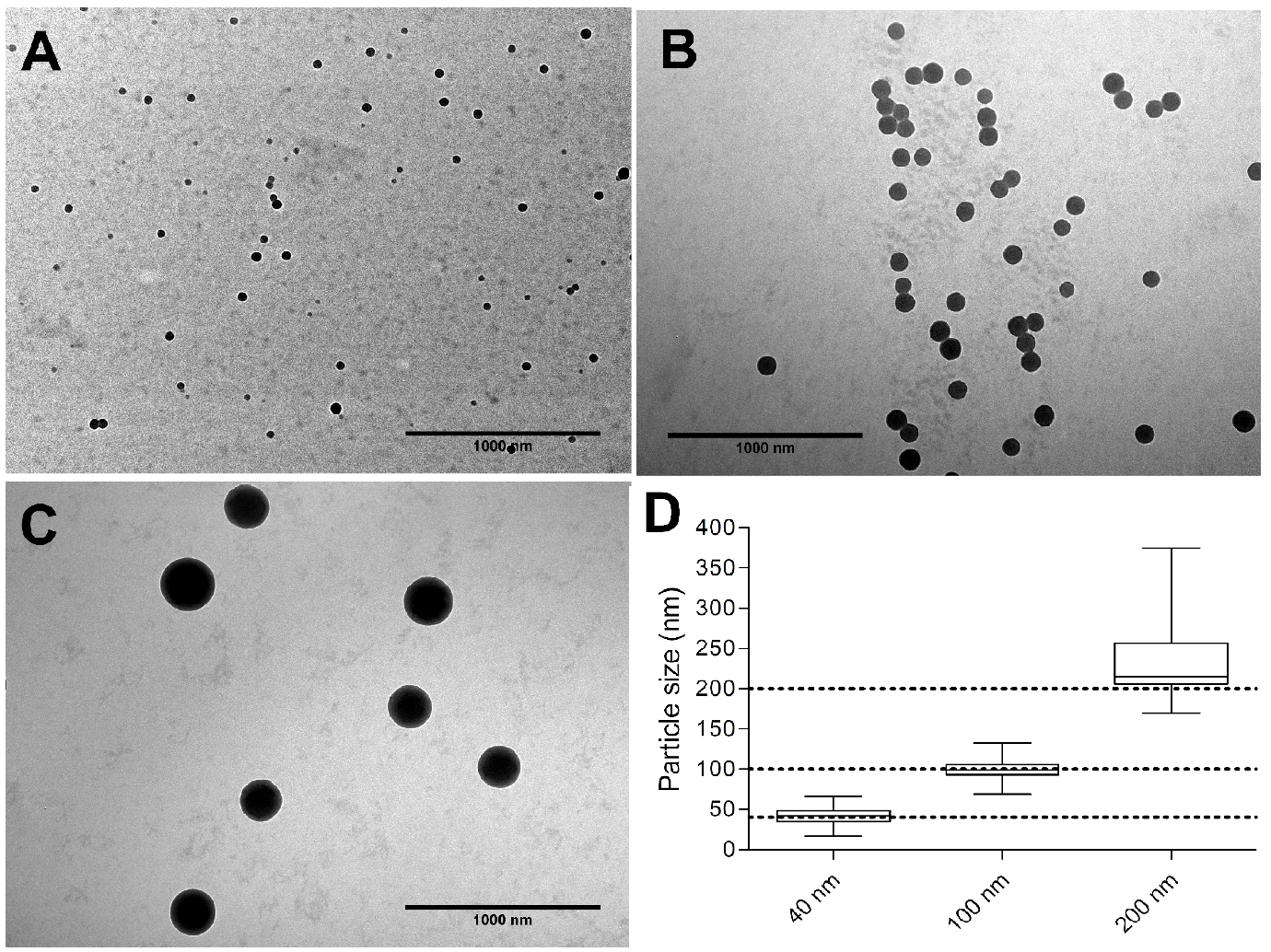
**

**Fig. S1:** Transmission electron microscopy images of 40 **(A)**, 100 **(B)** and 200 **(C)** nm PS NPs with magnification set at 36000. Boxplots in panel **(D)** demonstrate the mean size of at least 185 particles of the different PS NPs sizes, as well as minimum and maximum values of the data set.

**
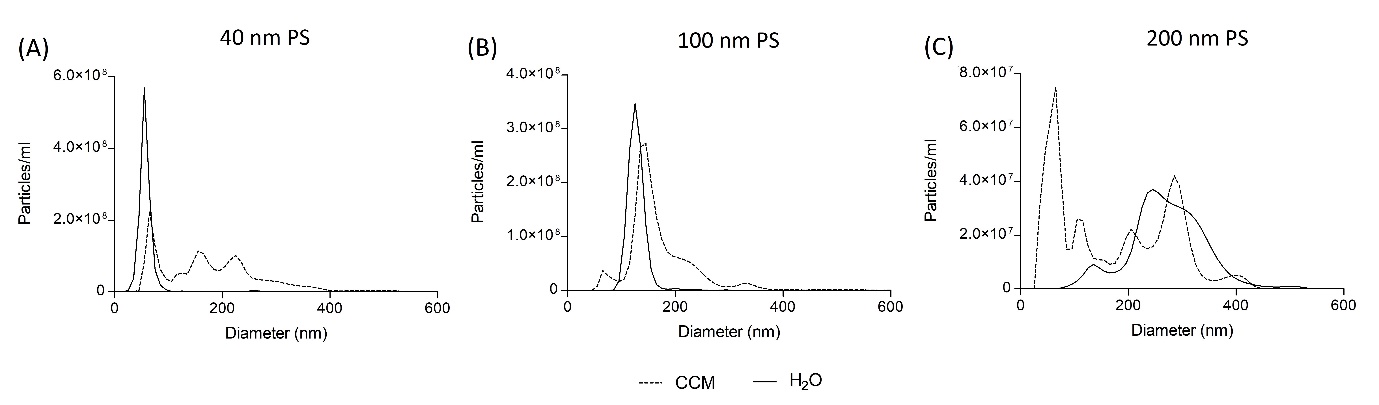
**

**Fig. S2:** Size distribution of 40 (A), 100 (B) and 200 (C) nm PS NPs in water and after 4 hours of incubation in serum-supplemented cell culture medium at 37°C and 5% CO_2_. Nanoparticle tracking analysis was used to measure the NPs’ hydrodynamic diameter, mean graphs of 3 replicate measurements are shown.

**
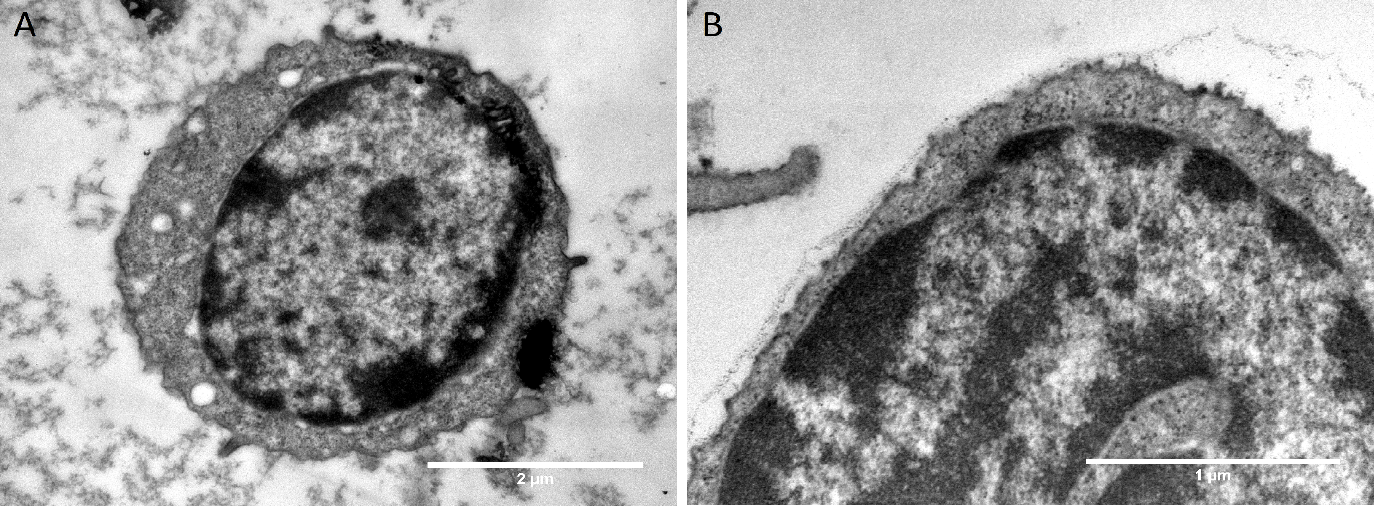
**

**Fig. S3:** Transmission electron microscopy images of HPCs stained with ruthenium with magnification set at 18000 **(A)** and 44000 **(B)**. Interpretation was done following Richardson et al., 1982^(1)^.

**
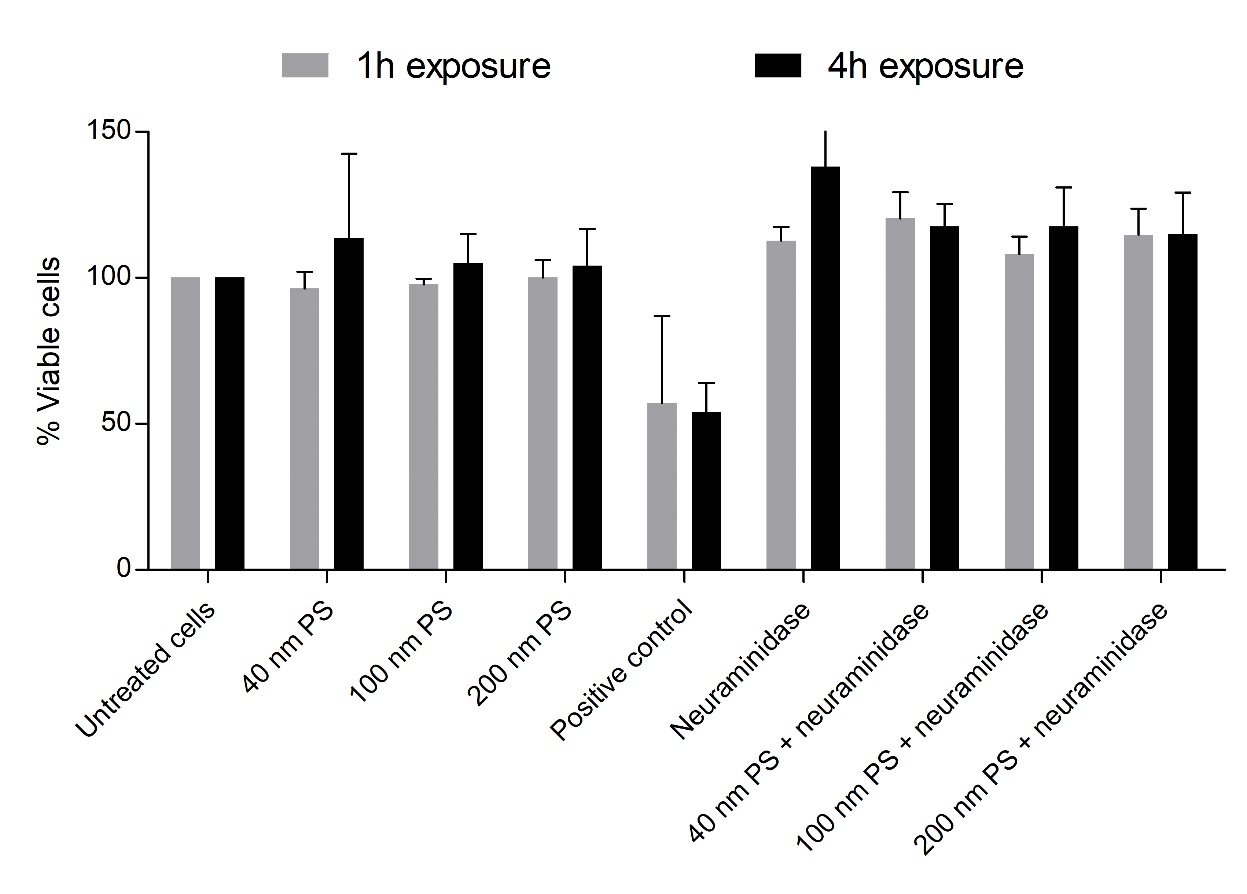
**

**Fig. S4:** Viability of THP-1 cells upon 1 and 4 hours of exposure to 50 µg/ml of 40, 100 and 200 nm PS NPs and/or 0.83 U/ml neuraminidase, measured by PrestoBlue assay. Staurosporine was used as a positive control in final concentrations of 50 µM for 1 hour and 10 µM for 4 hours. The mean % viable cells of 3 THP-1 cultures with different passage number are shown.

**
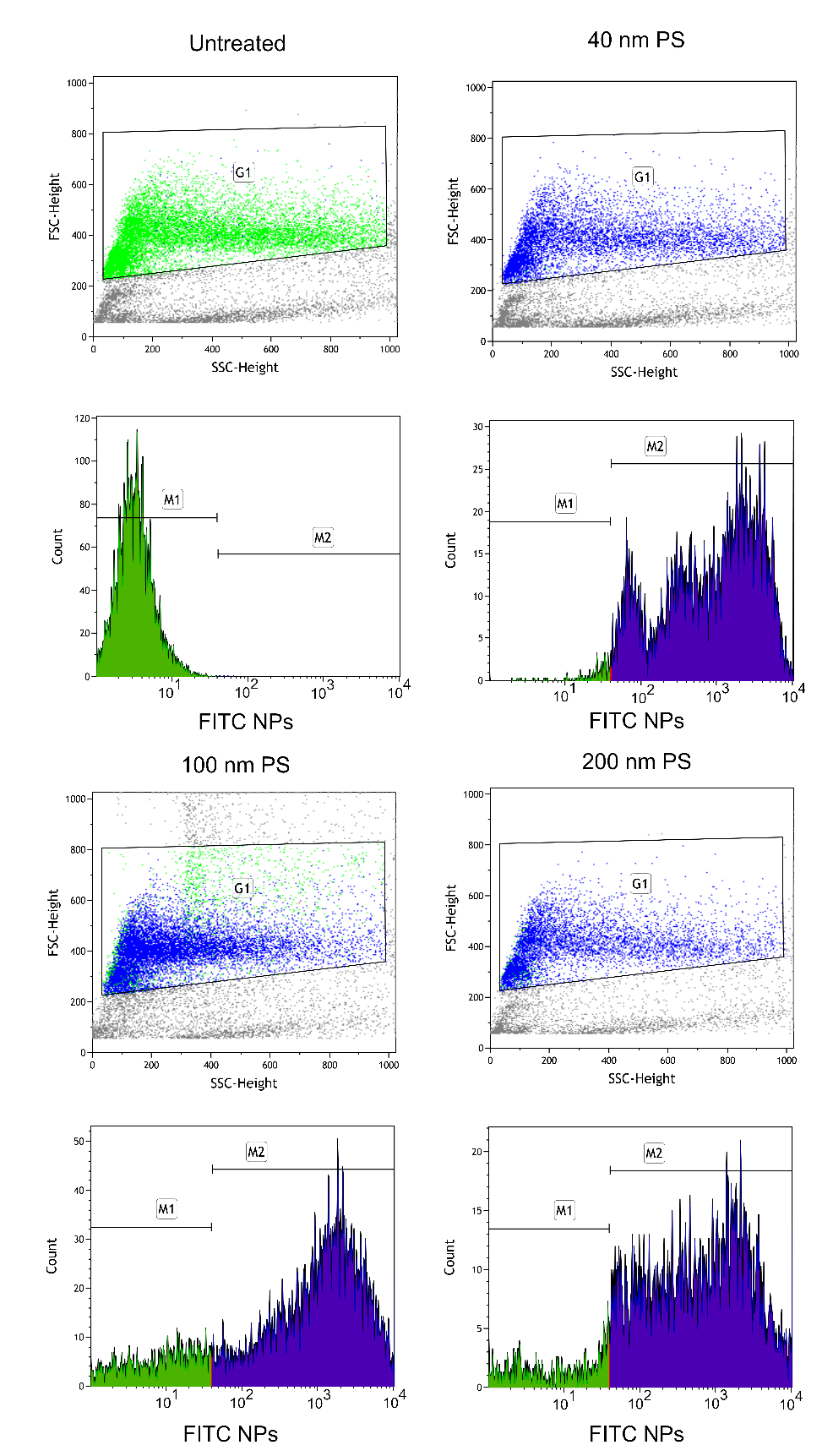
**

**Fig. S5:** Example scatter plots and fluorescence histogram plots of untreated HPC and HPCs exposed to 50 μg/ml of 40, 100 or 200 nm PS NPs for 1 hour. Gate G1 was set based on FSC and SSC to exclude cell debris. Markers were applied to the fluorescence histogram plots to distinguish blank cells (M1) from NP-loaded cells (M2). Marker bounds were set using untreated cell populations.

**
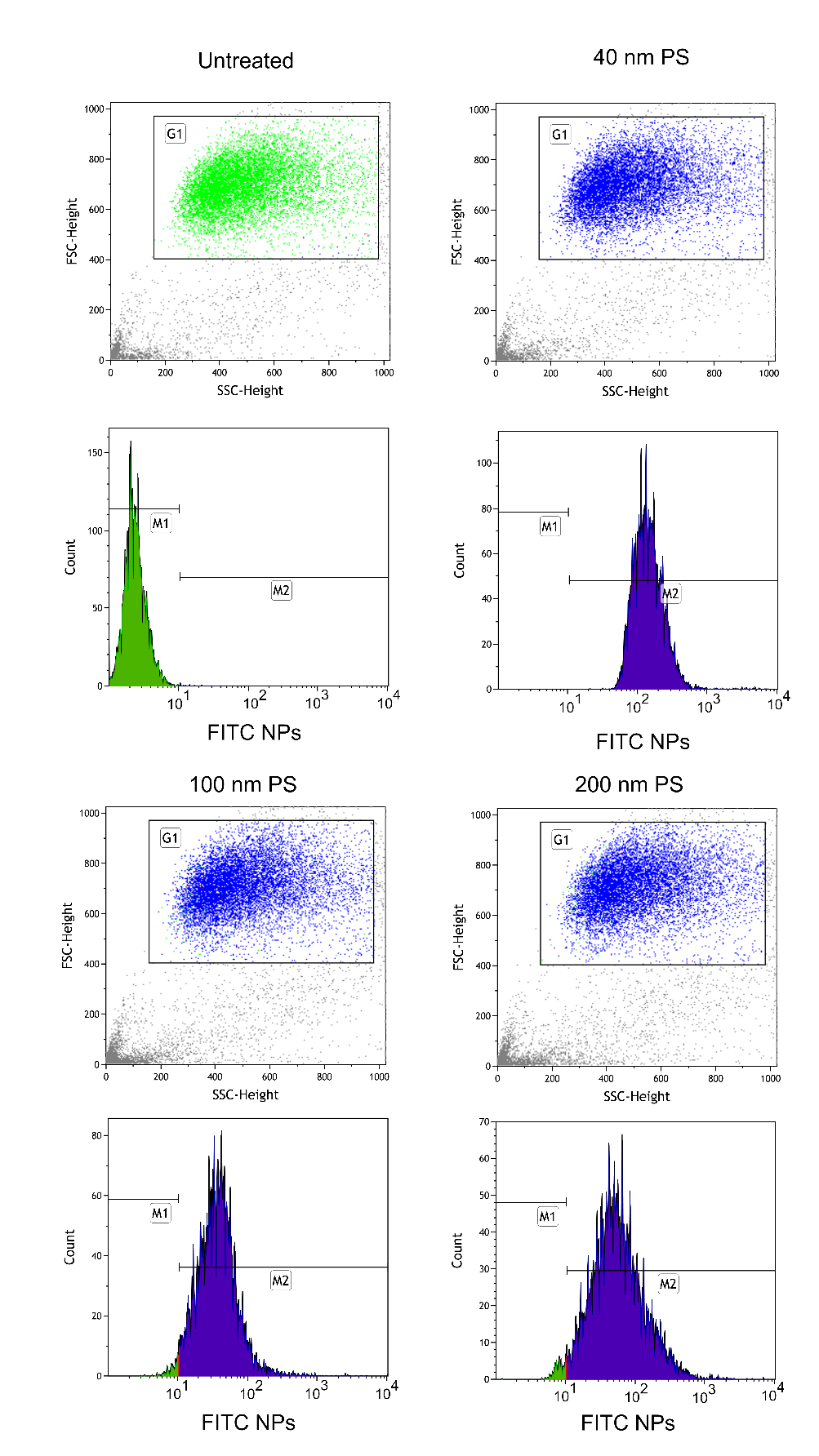
**

**Fig. S6:** Example scatter plots and fluorescence histogram plots of untreated THP-1 cells and THP-1 cells exposed to 50 μg/ml of 40, 100 or 200 nm PS NPs for 1 hour. Gate G1 was set based on FSC and SSC to exclude cell debris. Markers were applied to the fluorescence histogram plots to distinguish blank cells (M1) from NP-loaded cells (M2). Marker bounds were set using untreated cell populations.

# References

1. Richardson M, Gerrity RG, Alavi MZ, Moore S. Proteoglycan distribution in areas of differing permeability to Evans blue dye in the aortas of young pigs. An ultrastructural study. Arteriosclerosis (Dallas, Tex). 1982;2(5):369-79.
